# Chronic bronchitis and airflow obstruction is associated with household cooking fuel use among never-smoking women: a community-based cross-sectional study in Odisha, India

**DOI:** 10.1186/s12889-018-5846-2

**Published:** 2018-07-27

**Authors:** Asmi Panigrahi, Bijaya K. Padhi

**Affiliations:** 1 Rutgers New Jersey Medical School, Newark, NJ USA; 2 Center for Environmental and Occupational Health, AIPH University, Bhubaneswar, India

**Keywords:** Airflow obstruction, Chronic bronchitis, Solid biomass fuel, Household air pollution, PM2.5, Lung function, Liquefied petroleum gas

## Abstract

**Background:**

The use of solid biomass as cooking fuel could be a potential risk factor for chronic bronchitis (CB) and airflow obstruction (AFO) among never-smoking women. The disease burden in India among women is generally underestimated due to limited population-based epidemiological investigations. The aim of the study was to determine the prevalence of CB and AFO among never-smoking women, and its association with household cooking fuel use.

**Methods:**

We conducted a community-based cross-sectional study with a representative study sample (*N* = 1120) in Odisha, India during 2013–14. Study participants, never-smoking women aged 18–49 years, were recruited randomly from the population census. Trained community health volunteers administered a validated questionnaire that aligned with the standards of the Burden of Obstructive Lung Disease (BOLD) initiative and conducted spirometry. Prevalence estimates of CB (defined as “cough with productive of sputum for at least 3 months of the year for at least 2 years”) and AFO (pre-bronchodilator forced expiratory volume in 1 s (FEV1)/forced vital capacity (FVC) < 0.7) was estimated. Indoor PM2.5 exposure data were collected from a subset of 130 of the total 1120 homes in the study settings. Multivariable regression models were used to estimate the associated risk factors.

**Results:**

Prevalence of CB and AFO were 7.3 and 22.4% respectively among the study participants. Of the study participants, 31% used exclusive liquefied petroleum gas, 18% used mixed fuel and 51% exclusively used solid biomass fuel for household cooking. In adjusted analysis, both CB (odds ratio 1·96, 95% CI: 1.06–3.64; *p* = 0·031) and AFO (OR 5.55, 95% CI: 3.51–8.78; *p* < 0·001) were found to be associated with cooking with solid biomass fuel. Interquartile range increases in PM2.5 was associated with significantly lower FEV1/FVC ratio.

**Conclusions:**

The study highlights that the estimates of population burden of CB and AFO are much higher than shown in previous epidemiological studies, and that cooking fuel type and time spent on cooking were associated with increased chronic bronchitis as well as decreased lung function as measured by FEV1/FCV ratios. To most accurately understand the current burden of disease and most effectively prevent an escalation in the future disease burden, further epidemiological investigations are warranted.

## Background

Globally, chronic respiratory disease is a major public health problem [1]. A recent medical survey conducted in India concluded that 50% of patients report to clinics with respiratory problems [2]. Under the umbrella of lung diseases, chronic bronchitis (CB) has been shown to have the most significant clinical impact regarding both morbidity as well as the quality of life [3–6]. CB has been defined as chronic cough with sputum production for at least 3 months per year for two consecutive years, and varies across epidemiological investigations [7]. CB has been considered as a surrogate for a diagnosis of chronic respiratory diseases for epidemiological purposes [8]. The prevalence of CB in adults > 35 years or older in India has been reported to be 3.49% (4.29% in males and 2.7% in females) [9]. Lung function measurement by spirometry is the preferred modality for establishing an accurate diagnosis of airflow obstruction (AFO). However, empirical evidence of the burden of AFO in India, especially spirometry-based general population data, is lacking. Further, most healthcare facilities have limited pulmonary care expertise and clinical capacity to quantify the burden of AFO, and instead rely on symptomatic data reported by patients or their caregivers. With only a few exceptions, the result is that the existing estimates of mortality and morbidity related to AFO in India have been derived either from infrequent national hospital surveys, or extrapolation from statistical models [2, 9, 10]. Thus, while the significance and implications of AFO have been established, there remains a gap in measuring the true burden of disease in India.

Furthermore, much is known about the strategies for mitigating the disease burden [10], yet chronic respiratory diseases are still poised to emerge as a leading cause of morbidity and mortality in low-and-middle-income countries in the near future [11–14]. Though the primary risk factor for CB is smoking, numerous studies have been reporting CB among never-smokers, suggesting that other risk factors may exist [7, 15–18].

About 3 billion people worldwide, including 6.5 million Americans and nearly 700 million Indians, continue to use solid biomass fuel to meet their household energy needs [19–21]. Using solid fuels in open fires or stoves at home while cooking or heating produces extremely toxic pollutants including fumes containing a high concentration of inhalable particulate matters (PM) that can range from 300 to 3000 μg/m^3^, sometimes reaching levels as high as 10,000 μg/m^3^. This accounts for 4 million annual deaths worldwide [22–24]. Household air pollution resulting from combustion of solid biomass fuel could be a potential risk factor for CB and AFO among never smoking women, this relationship largely unexplored in India. We, therefore designed a community-based study to measure the prevalence of CB and AFO in an eastern state in India, as well as investigate the association between household cooking fuel use and the disease burden.

## Methods

### Study setting

This community-based cross-sectional study was carried out in the rural setting of Odisha, an eastern state in India, home to 41.9 million people. The state has one of the highest infant mortality (51 per 1000 live births) and maternal mortality (235 per 100,000 live births) rates in India [25]. The study was conducted in two blocks of Khordha districts (rural coastal settings), which have a population of about 200,000 individuals with approximately 30,000 women of reproductive age (13–49 years). Villages are densely populated and located at an altitude of 41.2 m. Inhabitants in the study site subsist on irrigated agriculture and work in government offices and other small service industries.

### Study design

**Fig. 1:**
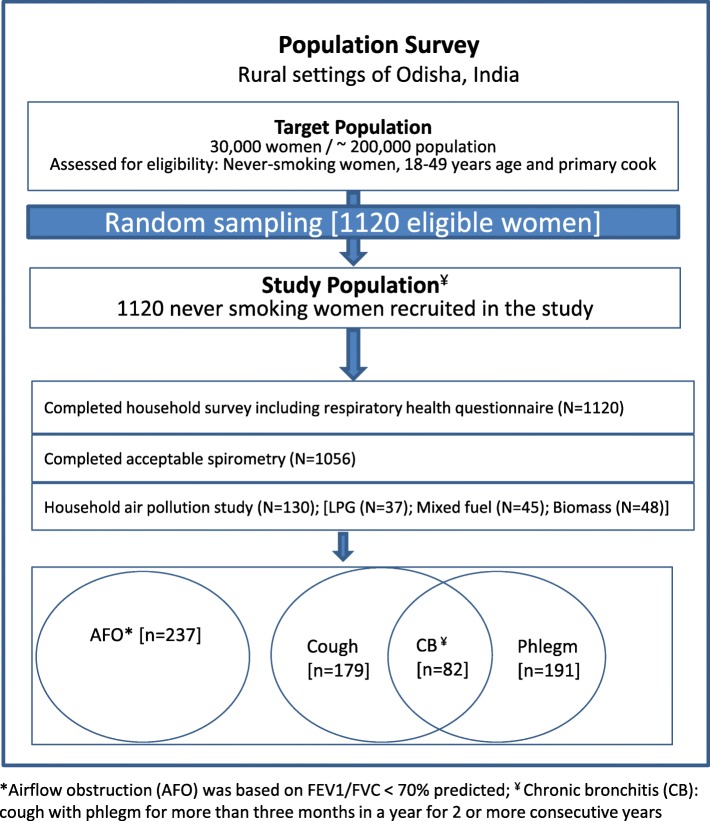
Schematic representation of sampling scheme and study findings

A community-based cross-sectional study was designed based on a priori hypothesis, and the study participants, never-smoking women and primary cook in the household, aged 18–49 years were recruited randomly from the population census developed by the study institute (Asian Institute of Public Health) (Fig. 1). The sample size of 1117 was estimated to obtain a 95% confidence interval (CI) of ±1.0% around a prevalence estimate of 3% [9]. We recruited 1120 individuals, anticipating some non-response among the study participants for detailed household survey, including lung function measurements and respiratory symptoms assessment. The sample size was calculated using the OpenEpi tools [26].

### Data collection tools

Our study is in accordance with the Burden of Obstructive Lung Disease (BOLD) initiative [8]. We used the International Union Against Tuberculosis and Lung Disease (IUATLD) English questionnaire [27] to study the prevalence of chronic airflow diseases in the study population. The same questionnaire has been validated and tested for its reliability in the “Indian study on epidemiology of asthma, respiratory symptoms and chronic bronchitis in adults (INSEARCH)” in the local context [9]. The study questionnaire included questions on respiratory disease symptoms as well as questions on socio-demographics and environmental exposures. The questions administered covered study participants’ history of chronic bronchitis (cough and phlegm for ≥3 months of the year for ≥2 years), chronic cough (with/without phlegm) and chronic phlegm (with/without cough). Based on household cooking fuel preferences, we categorized the households into three groups “LPG: households used exclusively Liquefied Petroleum Gas (LPG) for cooking”; “mixed fuel: household used a combination of fuel (LPG, kerosene, electricity, and solid biomass) for cooking” and; “solid biomass fuel: household used exclusively biomass fuel for cooking”.

Our primary outcome measure was to study the prevalence of CB and AFO. We diagnosed CB in the study population on the basis of affirmative responses to the IUATLD questions, which consisted of the presence of cough with expectoration for more than 3 months in a year for two or more consecutive years [9, 27].

### Spirometry and definition of AFO

Spirometric testing was carried out using a MicroLoop spirometer (CareFusion, USA) by a trained community health supervisor and conducted in accordance with American Thoracic Society/ European Respiratory Society guidelines. All spirometry was conducted at the household of the study participants and performed during the day time (9:00 AM-2:00 PM). Participants were instructed to breathe in and out through the mouthpiece as deeply and quickly as possible while a nose clip was applied in a sitting position. We used south Indian reference values for spirometry [28]. The spirometer acquired the forced vital capacity (FVC), forced expiratory volume in 1 sec (FEV1), and the ratio of (FEV1)/(FVC). The device records the best performance of the three successful/acceptable efforts. Since post-bronchodilator measurements were not obtained we categorised AFO status using the modified Global Initiative for Chronic Obstructive Lung Disease (GOLD) criteria (GOLD grade ≥ 1: FEV1/FVC < 0.7; GOLD ≥2: FEV1/FVC < 0.7 and FEV1/predicted FEV1 < 0.8; GOLD ≥3: FEV1/FVC < 0.7 and FEV1/predicted FEV1 < 0.5) [29]. Participant’s age, height, and weight were collected at the time of spirometry. Weight was measured using an electronic balance (calibrated daily), and height was recorded using a metal tape with height mark on the wall. Of the 1120 women who participated in the survey, 1056 could perform the spirometry adequately for further analysis.

### Environmental exposure monitoring

Household PM2.5 exposure assessment was conducted in a subset (*N* = 130) of the study households selected conveniently according to the household cooking behavior. PM2.5 was monitored using Minivol (Airmatrix) sampler operated at 10 L/min and collected particles on a 47 mm quartz filter (Whatman International, Ltd., Maidstone, England). Before mounting the filter paper, the serial number was recorded and the filter paper equilibrated overnight in ambient temperature and humidity. A calibrated micro-balance (Mettler Instrument Corp., Hightstown, NJ) was used to weigh the filter papers with a precision of ±5.0 μg. A thorough quality inspection was conducted to identify any tears, folds, and other imperfections. Sampling was conducted at the center of the living room and placed 1.5 m above the ground, and at least 0.5 m away from walls. After sampling, the particle-loaded filter papers were placed in a sealed container and transported to the laboratory for further analysis. Total mass concentration of each filter was estimated by weighing the particle-loaded filters following a 24 h equilibration period. The sampling was carried out for 12 h (8.0 am - 8.0 pm) and monitored only once in the study households.

### Data analysis

A database was created using a custom-designed Epi-info platform. Descriptive statistics (frequency, means, and standard deviations) were calculated and cross-tabulated with household fuel use. Group comparisons were performed using the *chi-square* (χ^2^) test (for categorical variables) and ANOVA (for continuous variables). Principal component analysis with varimax rotation was used for computing a socio-economic status (SES) index [30] from the household characteristics and asset data. Based on the distribution of the SES indices, the households were then divided into three groups (tertiles) of socio-economic status: low, medium, and high.

Unadjusted and adjusted odds ratios and 95% CIs were computed using logistic regression models to estimate the associated risk factors. All multivariable regression models were performed using a priori hypotheses. Results from all analyses were considered significant at a *p*-value of 0.05. All data analysis including production of tables and figures were performed using Stata® Software version SE 13.0 (College Station, TX, USA).

## Results

All 1120 participants completed the survey. Of the 1120, spirometry could be adequately performed in 1056 participants. Table 1 shows the characteristics of the participants stratified by participant’s household cooking fuel types. Of the study participants only 31% of women cooked exclusively with a cleaner fuel (LPG), approximately half of them used solid biomass fuel (51%), and 18% had used mixed fuel (LPG, electricity, and solid biomass). The mean age of participants was 30 years, and the mean BMI was 23.3 ± 3.5. About 6.8% of the solid biomass users and 10% of total study participants were from higher socioeconomic status. Most of the participants completed their primary education (96.5%) with less variation across groups. Ninety-four percent of the respondents were homemakers, and 66% of them were married. 5.6% of participants were exposed to environmental tobacco smoke in childhood or adulthood. Only 12% households had a separate kitchen, and only 16% of the kitchens were ventilated. One percent of participants had a family history of asthma.

**Table 1:** Socio-demographic characteristics of the Study Population (*N* = 1120)

Characteristics	All participants (*N* = 1120)	Liquefied petroleum gas user (*N* = 344)	Mixed fuel user (*N* = 203)	Solid biomass fuel user (*N* = 573)	*p*-value
Anthropometrics: Age (years), Height (cm) and Weight (Kg)					
Age (mean ± SD)	30.44 ± 6.86	31.06 ± 6.92	31.64 ± 7.26	29.63 ± 6.58	< 0.001
Height (mean ± SD)	154.67 ± 8.35	152.83 ± 8.04	155.48 ± 7.86	155.48 ± 8.54	< 0.001
Weight (mean ± SD)	55.50 ± 7.67	53.84 ± 7.79	55.33 ± 7.50	56.56 ± 7.49	< 0.001
BMI (mean ± SD)	23.30 ± 3.50	23.14 ± 3.49	22.96 ± 3.26	23.53 ± 3.57	0.079
Marital Status [n, %]					
Married	745 (66.5)	228 (66.2)	138 (67.9)	379 (66.1)	0.887
Unmarried	375 (33.4)	116 (33.7)	65 (32.0)	194 (33.8)	
Socio-economic status (SES) Indices^a^ [n, %]					
Low	114 (10.18)	8 (2.33)	22 (10.84)	84 (14.66)	< 0.001
Middle	895 (79.91)	279 (81.10)	166 (81.77)	450 (78.53)	
High	111 (9.91)	57 (16.57)	15 (7.39)	39 (6.81)	
Education level [n, %]					
No education	16 (1.43)	3 (0.87)	4 (1.97)	9 (1.57)	0.094
Primary	1081 (96.52)	330 (96.24)	197 (97.04)	554 (96.68)	
Secondary/college	23 (2.05)	11 (3.19)	2 (0.98)	10 (1.75)	
Occupation [n, %]					
Office worker	29 (2.59)	12 (3.49)	4 (1.97)	13 (2.27)	0.026
Housewife	1053 (94.02)	312 (90.70)	193 (95.07)	584 (95.64)	
Other	38 (3.39)	20 (5.81)	6 (2.96)	12 (2.09)	
Asthma in family [n, %]					
No	1105 (98.66)	339 (98.55)	197 (97.04)	569 (99.30)	0.054
Yes	15 (1.34)	5 (1.45)	6 (2.96)	4 (0.70)	
Cooking hours and age [mean ± SD]					
Cooking hours per day	4.23 ± 1.10	2.31 ± 0.62	4.04 ± 1.08	4.45 ± 0.80	< 0.001
Years cooking	9.97 ± 5.12	9.13 ± 5.28	11.28 ± 6.04	10.01 ± 4.54	< 0.001
Separate Kitchen [n, %]					
Yes	142 (12.67)	70 (20.34)	17 (8.37)	55 (9.59)	< 0.001
Ventilated kitchen [n, %]					
Yes	186 (16.60)	90 (26.16)	25 (12.31)	71 (12.39)	< 0.001
Environmental tobacco smoke (ETS) exposure [n, %]					
None	994 (88.75)	309 (89.82)	180 (88.66)	505 (88.13)	0.002
Childhood	32 (2.85)	15 (4.36)	5 (2.46)	12 (2.09)	
Adulthood	31 (2.76)	14 (4.06)	3 (1.47)	14 (2.44)	
Both	63 (5.62)	6 (1.74)	15 (7.38)	42 (7.32)	

^a^Principal component analysis with varimax rotation was used for computing the SES indices. Based on the distribution of the SES indices, the households were then divided into three groups (tertiles)

**Fig. 2:**
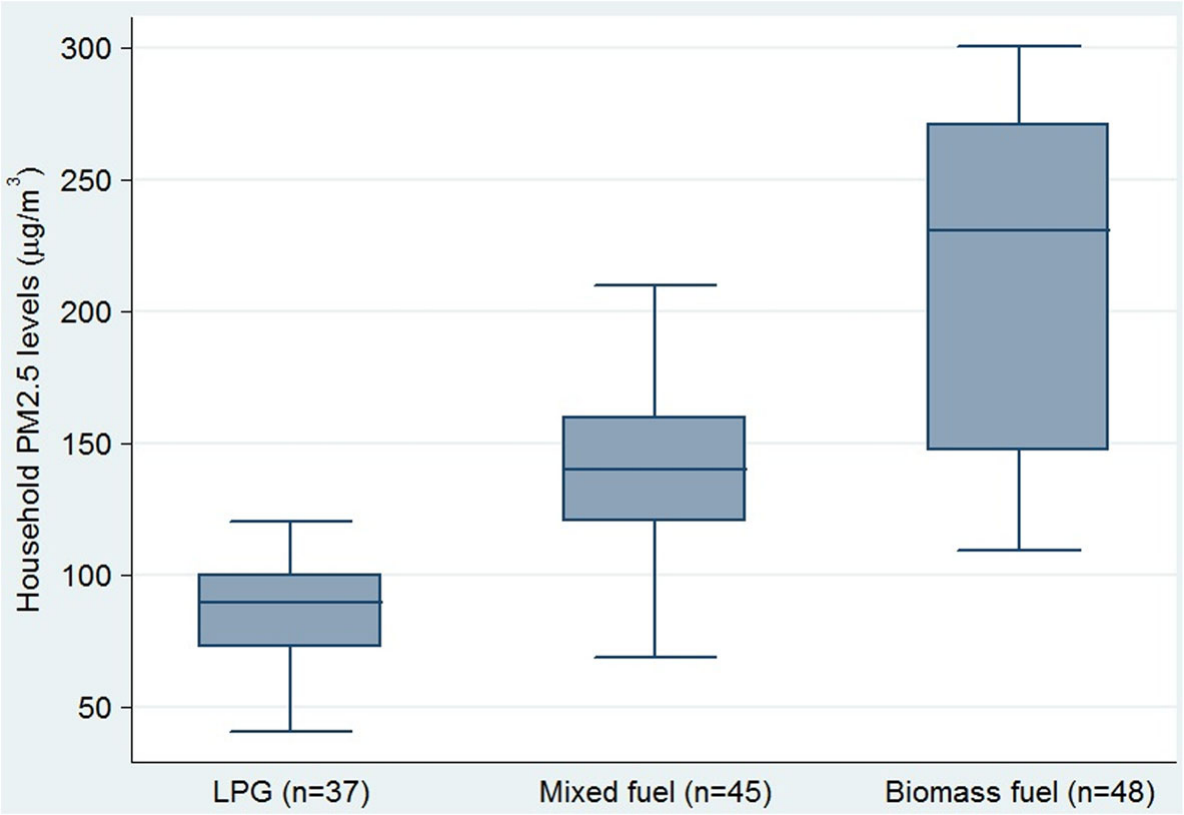
Box plots comparing average PM2.5 levels in homes (*N* = 130) stratified by cooking fuels: LPG (*n* = 37); Mixed (*n* = 45) and Solid biomass (*n* = 48). [Boxes represent the interquartile range (25th–75th percentiles, median indicated by horizontal line), and whiskers extend to the 5th and 95th percentiles]

The 5th to 95th percentile ranges of the household exposure PM2.5 distribution is shown in Fig. 2, ranging from 40 to 300 μg/m^3^ (vs. mean of 148.6 μg/m^3^), demonstrating the considerable variation in fine particle exposure across the households. The median PM2.5 levels were 87.8 μg/m^3^, 132.2 μg/m^3^, and 230.6 μg/m^3^ in LPG, mixed fuel and solid biomass fuel burning household respectively (Fig. 2).

The prevalence of chronic bronchitis, including other respiratory symptoms, is shown in Table 2. The overall prevalence of chronic bronchitis was 7.3% (82 of the 1120 participants). The CB prevalence was lower (*n* = 15, 4.3%) among LPG users than mixed fuel (*n* = 17, 8.3%) and solid biomass fuel (*n* = 50, 8.7%) user groups. A significant difference of prevalence of wheezing was observed across study groups and was higher among biomass users (12.7%).

**Table 2:** Prevalence of self-reported respiratory symptoms among respondents stratified by household fuel use

Respiratory symptoms^a^	All Participants (*N* = 1120)	Liquefied petroleum gas user (*n* = 344)	Mixed fuel user (*n* = 203)	Solid biomass fuel user (*n* = 573)	*p*-value
Wheeze	114 (10.18)	22 (6.40)	19 (9.36)	73 (12.74)	0.008
Cough at night	138 (12.32)	43 (12.50)	21 (10.34)	74 (12.91)	0.628
Cough in morning	179 (15.98)	55 (15.99)	29 (14.29)	95 (16.58)	0.746
Phlegm in morning	191 (17.05)	57 (16.57)	30 (14.78)	104 (18.15)	0.525
Chronic bronchitis^b^	82 (7.32)	15 (4.36)	17 (8.37)	50 (8.73)	0.040
Morning breathlessness	189 (16.88)	48 (13.95)	42 (20.69)	99 (17.28)	0.119
Breathlessness on exertion	172 (15.36)	49 (14.24)	38 (18.72)	85 (14.83)	0.331
Chest tightness on dust exposure	206 (18.39)	55 (15.99)	33 (16.26)	118 (20.59)	0.150
Physician diagnosed asthma^c^	59 (5.27)	12 (3.49)	13 (6.40)	34 (5.93)	0.200

^a^Values reported in table are n (%)

^b^Chronic bronchitis: cough with phlegm for more than 3 months in a year for 2 or more consecutive years

^c^Physician-diagnosed asthma was defined as participants had been diagnosed with asthma and use of anti-asthmatic medication

Table 3 presents the analysis of multivariable logistic regression model. Household cooking fuel use was found to be associated with CB in the multivariable model. Solid biomass fuel users had a significantly higher risk (AOR = 1.96; 95% CI: 1.06–3.64) of CB. Households practicing cooking with mixed fuel were not found to have any protective effect against CB (AOR = 1.85; 95% CI: 0.87–3.89).

**Table 3:** Multiple logistic regression analysis of association between household fuel use and chronic bronchitis (*N* = 1120)

Household fuel use	Odds ratios (95% CI)		*p* value
	Unadjusted	Adjusted^a^	
Liquefied petroleum gas	1 (reference)	1 (reference)	
Mixed fuel	2.00 (0.97–4.10)	1.85 (0.87–3.89)	0.105
Solid biomass fuel	2.09 (1.15–3.79)	1.96 (1.06–3.64)	0.031

^a^Adjusted for age, BMI, socio-economics status, and education

The prevalence of AFOs are shown in Table 4 by current cooking fuel. Of the 1056 participants who provided acceptable spirometry, 22.4% of them were found to be at risk of AFO (FEV1/FVC < 70% predicted). The extent of obstruction was higher among biomass fuel users (31%).

**Table 4:** Prevalence of airway obstruction based on spirometry performance among respondents stratified by household fuel use (*N* = 1056)

Lung function indices^a^	All Participants (*N* = 1056)	Liquefied petroleum gas user (*n* = 319)	Mixed fuel user (*n* = 202)	Solid biomass fuel user (*n* = 535)	*p*-value
FEV1/FVC < 70% predicted	237 (22.4)	25 (7.84)	46 (22.77)	166 (31.03)	< 0.001
FEV1/FVC < 70% and FEV1 < 80% predicted	103 (9.75)	9 (2.82)	21 (10.4)	73 (13.64)	< 0.001
FEV1/FVC < 70% and FEV1 < 50% predicted	23 (2.17)	2 (0.18)	6 (0.56)	15 (1.42)	< 0.001

^a^Values reported in table are n (%)

Table 5 shows odds ratios for associations between AFO and household cooking fuel use. In regression models adjusted by age, BMI, education, and socio-economic status, solid biomass fuel use was associated with a 5.5% decrease in FEV1/FVC ratio (AOR = 5.55; 95% CI: 3.51–8.78).

**Table 5:** Multiple logistic regression analysis of association between household fuel use and airflow obstruction (*N* = 1056)

Household fuel use	Odds ratios (95% CI)		*p* value
	Unadjusted	Adjusted^a^	
Liquefied petroleum gas	1 (reference)	1 (reference)	
Mixed fuel	3.46 (2.05–5.85)	3.47 (2.04–5.90)	< 0.001
Solid biomass fuel	5.29 (3.38–8.27)	5.55 (3.51–8.78)	< 0.001

^a^Adjusted for age, BMI, socio-economics status, and education

Table 6 shows the association between FEV1/FVC ratio with household PM2.5 levels and respondents cooking behavior in a robust multivariable regression model. An interquartile increase in PM2.5 increased the probability of having a low FEV1/FVC ratio by 4.4 percentage points and the ratio declines by 7% when the PM2.5 levels were above 196.8 μg/m^3^ (> 75th percentile). Respondents having higher cooking ages had 6.9% lower FEV1/FVC ratio than participants engaged in cooking for less number of years. Similarly, those cooking for four and half hours or more in a day showed a decline in FEV1/FVC ratio by 6.9% (Table 6). The study showed a strong association between household air pollution (PM2.5 levels) and AFO (lower FEV1/FVC ratio) (Table 6).

**Table 6:** Multivariable robust regression models for predictors of lung function indices (FEV1/FVC ratio) (*N* = 1056)

Predictors	β coefficients (95% CI)		*p* value
	Unadjusted	Adjusted^a^	
Household PM2.5 (μg/m^3^)			
Low (< 92.4 μg/m^3^)	0 (reference)	0 (reference)	
[<25th percentile]			
High (92.4–196.8 μg/m^3^)	− 0.098 (− 0.128 - -0.069)	− 0.044 (− 0.074 - -0.013)	0.005
[25th – 75th percentile]			
Higher (> 196.8 μg/m^3^)	−0.117 (− 0.166 - -0.068)	−0.070 (− 0.124 - -0.015)	0.013
[> 75th percentile]			
Cooking age			
< 5 years	0 (reference)	0 (reference)	
5–15 years	−0.082 (− 0.093 - -0.071)	−0.028 (− 0.055 - -0.000)	0.044
Above 15 years	− 0.122 (− 0.142 - -0.102)	−0.069 (− 0.120 - -0.019)	0.007
Cooking hours per day			
< 2.5 h	0 (reference)	0 (reference)	
2.5–4 h	−0.047 (− 0.058 - -0.036)	−0.037 (− 0.068 - -0.006)	0.020
> 4 h	−0.108 (− 0.120 - -0.097)	−0.069 (− 0.112 - -0.025)	0.002
Environmental tobacco smoke (ETS) Exposure			
None	0 (reference)	0 (reference)	
Childhood	−0.003 (− 0.031 - -0.024)	−0.012 (− 0.051–0.025)	0.510
Adulthood	−0.021 (− 0.070 - -0.026)	0.040 (− 0.018–0.100)	0.177
Both	−0.151 (− 0.177 - -0.125)	−0.063 (− 0.123 - -0.002)	0.041

^a^Adjusted for age, BMI, marital status, education, socio-economic status, kitchen ventilation

## Discussions

To our knowledge, this is the first population-based epidemiological study in Odisha, India to report on the relationship between household cooking behavior and household air quality and its association with CB as well as with AFO using spirometry data. We also demonstrated a significant decline in lung function with exposure to higher household PM2.5 concentration among never-smoking women in India. The association was robust and insensitive to potential confounders such as age, BMI, education and socio-economic status.

Prevalence of chronic bronchitis in our study was nearly twice than that reported in the earlier nationwide Indian study (INSEARCH), conducted in hospital settings with a reported prevalence of chronic bronchitis of 2.7% among women older than 35 years [9]. A significant finding of our study is that the overall prevalence of AFO on spirometry is 22.4% (nearly one-fifth of the total study population). Such a finding has considerable implications, highlighting the phenomenal burden of obstructive lung disease in India likely obscured by what has been, until this study, a lack of accurate epidemiological measurement methods. As demonstrated by the study results, the definition of CB has a weak correlation with the prevalence of spirometric evidence of AFO and underestimates the true prevalence of obstructive lung diseases in the community. As a formal physician assessment was not involved in the study, we cannot derive the exact diagnoses in the non-CB patients. Nevertheless, these findings lay the groundwork for further research in understanding the accurate epidemiology of obstructive lung diseases in India. Another significant finding that is highlighted is the fact that spirometry must be incorporated as an essential investigation tool in field epidemiological studies of obstructive lung diseases in order to understand the true burden of the problem. We plan to undertake a detailed physician-based assessment of the patients with evidence of AFO on spirometry so that the follow-up data in this regard can provide us useful information in the future.

In our study, as expected, household PM2.5 particle concentrations were higher in homes using biomass fuel than in homes using LPG (Fig. 2). The average PM2.5 levels measured in the living room of the study participants using solid biomass fuel for cooking were three times greater than those of LPG burning households (81.5 μg /m^3^). The mixed fuel users were also found to be exposed to higher PM2.5 in the household air (134.2 μg/m^3^). Our findings are similar to previous reports of household PM2.5 particle concentrations from biomass fuel use [31–34]. In Nepal, the average PM2.5 concentration in biomass burning kitchens was found to be 656 μg/m^3^ [31]. In China, biomass fuel burning is contributed to personal average 24-h exposure to PM2.5 ranged from 22 to 634 μg/m^3^ in winter and from 9 to 492 μg/m3 in summer [32]. Another study in China reported personal exposures to PM2.5 concentrations ranging (Geometric mean) 225–289 μg/m^3^ during the burning of wood or plant materials. In Indian households that used solid fuels, the mean 24-h concentration of PM2.5 was found to be 163 μg/m^3^ (95% CI: 143,183; median 106; IQR: 191) in the living area.

Our reported prevalence of chronic bronchitis among women is in the range of other reports in India and elsewhere. In our study, the risk of chronic bronchitis among biomass fuel users was significantly higher than in users of LPG or mixed fuel. An Indian study in southern parts of the country reported that biomass fuel users have a higher risk of COPD than the clean fuel users at 2.5% vs. 2% [35]. Another study conducted in eastern parts of India reported that women who used biomass fuel are reported to have a higher risk of COPD than LPG fuel users at 4.6% vs 0.9% [36].

The biomass fuel users had a lower FEV1/FVC ratio in our study population. We observed a statistically significant association of household PM2.5 concentration and change in lung function indices. A qualitative comparison can be made with other studies which have reported that exposure to biomass smoke is associated with reduced lung function and respiratory symptoms as seen in Guatemala [13, 37], Brazil [38], Malawi [39], Mexico [16], and India [35, 36]. A randomized exposure study in Guatemala reported CO in exhaled breath was associated with lower lung function in FEV1. The study found that a 10% increase in CO was associated with 3.33 mL of FEV1 [37]. The Mexican study reported that biomass fuel was associated with increased production of phlegm (27 vs. 9%) and reduced FEV1/FVC ratio (79.9 vs. 82.8%) [16]. The study also highlighted that in homes with higher PM10 concentration, cough was more common among women who had lower values of FEV1 (odds ratio, 1.7; 95% confidence interval, 1.0–2.8).

A critical finding of our study is the high prevalence (237 of 1120 participants, 22.4%) of spirometric evidence of AFO in the study population. This highlights that overall nearly one-fifth of the population has obstructive airflow disease, and thus this work raises important questions about most epidemiological studies that have used symptomatic diagnostic criteria for quantifying the burden of lung diseases in India and elsewhere. AFO was found to be greater among solid biomass fuel users (31%), as approximately one-third of the population with the exclusive cooking use of solid biomass demonstrated spirometric evidence of AFO, indicating that solid biomass use could be a strong risk factor for chronic lung diseases in the Indian population. Given the extent of AFO revealed with spirometric measurement, the study findings suggest that use of only a questionnaire-based assessment of respiratory symptoms grossly underestimates the presence of obstructive lung disease in the Indian population.

We acknowledge some limitations to our study. First, the study is cross-sectional in nature and there could be recall bias in estimation of CB. Second, there is room for improvement in PM2.5 exposure assessment, because we have only accounted for indoor exposures in the living room. Third, we did not account for seasonal variation of both household exposure and outcome assessments. Another limitation includes the non-performance of post-bronchodilator spirometry, as a large difference between spirometry-measured airflow obstruction (FEV1/FVC ratio < 0.70) and self-reported chronic bronchitis is partly attributable to failure to exclude subjects with non-COPD-related airway obstruction like asthma. A proportion of the participants with spirometric evidence of obstruction may have reversible AFO, signifying the presence of bronchial asthma rather than fixed AFO indicative of COPD. Lastly, we cannot rule out any residual or unmeasured confounding variables in this study.

## Conclusions

The role of household air pollution exposures on health outcomes including CB and AFO among never-smoking women is poorly documented. A significant association was observed between environmental exposures (household PM2.5) levels and change in lung function among women in India. Given the challenges in diagnosing lung diseases early in resource-poor settings, our findings have significant public health implications. Our study findings, based on a robust representative sample in resource-poor settings, using a priori specified analyses contribute to strengthening the evidence on the association of environmental exposures and AFO. Never-smoking women exposed to solid biomass smoke reported lower lung function and more frequent cough and phlegm production than did women cooking with LPG.

## Abbreviations

AFO: Airflow obstruction
BOLD: Burden of obstructive lung disease
CB: Chronic bronchitis
CHV: Community health volunteers
COPD: Chronic obstructive lung disease
ETS: Environmental tobacco smoke
FEV1: Forced expiratory volume in one second
FVC: Forced vital capacity
IUATLD: International union against tuberculosis and lung disease
LPG: Liquified petroleum gas
PM: Particulate matter
SES: Socio-economic status
